# Supplementary material for: Content-rich biological network constructed by mining PubMed abstracts
Source: BMC Bioinformatics. 2004 Oct 8;5:147. doi: 10.1186/1471-2105-5-147 (PMC528731; doi:10.1186/1471-2105-5-147)
Supplement: Additional File 2 — The original results of the above study (non-essential files are deleted to keep the file size under the limit set by BMC bioinformatics). [file 1471-2105-5-147-S2.bz2 › chilibotAdditionalFile2/dip10/565ID10574949E10926/html/FGF9_FGFR1.html]

 


 **FGF9** and **FGFR1** 
  
Found 17 abstracts in PubMed, retrieved 10.  
 

 What does Google say? 
 PDF only 
| .edu only 

---

**Interactive relationship** (e.g. stimulation, inhibition, etc)

**Neutral relationship**- FGF 9  [ **FGF9** ]  altered FGFR  [ **FGFR1** ]  and myelin protein levels during OL differentiation.  Ref: 10900074 J Neurosci Res, 2000
- **FGFR1**  bound well to FGF2 but poorly to FGF8 and  **FGF9** .  Ref: 10574949 J Biol Chem, 1999
- Since receptor activation can result in its movement to a perinuclear localization, an alternative explanation for the redistribution of FGFR  [ **FGFR1** ]  3 IIIb could be different degrees of activation by a ligand FGF1 or  **FGF9** .  Ref: 11329138 J Pathol, 2001

**Non-interactive relationship** (e.g. studied together, co-existance, homology, etc.)

- some examples are avian erythroblastic leukemia viral oncogene homolog 3, neuregulin 1, fibroblast growth factor 9  [ **FGF9** ] , and fibroblast growth factor receptor 1  [ **FGFR1** ] .  Ref: 12021921 Virchows Arch, 2002
- Expression of each FGFR  [ **FGFR1** ]  and effects of the preferred ligand for FGFR  [ **FGFR1** ]  3,  **FGF9** , upon RGC survival and neurite outgrowth were examined in primary retinal cell cultures whereas there was no stimulation of neuritogenesis, RGC survival was promoted in a dose dependent manner ED 50 approximately 500 pg ml, mean maximal increase of 60% and could be completely blocked by addition of  **FGF9**  neutralising antibody.  Ref: 12799136 Mol Cell Neurosci, 2003
- The effects of FGF 9  [ **FGF9** ]  and FGF 2 on FGFR  [ **FGFR1** ]  and myelin protein levels were comparable.  Ref: 10900074 J Neurosci Res, 2000
- Taken together, these data suggest that the ligand receptor couple  **FGF9**  FGFR  [ **FGFR1** ]  3 may function to promote survival of adult mammalian RGC, and their application might be beneficial in retinal degenerative diseases such as glaucoma.  Ref: 12799136 Mol Cell Neurosci, 2003
